# Tailor-made thermoplastic elastomers: customisable materials *via* modulation of molecular weight distributions†

**DOI:** 10.1039/c9sc05278j

**Published:** 2019-12-17

**Authors:** Stephanie I. Rosenbloom, Dillon T. Gentekos, Meredith N. Silberstein, Brett P. Fors

**Affiliations:** Department of Chemistry and Chemical Biology, Cornell University Ithaca New York 14853 USA bpf46@cornell.edu; Sibley School of Mechanical and Aerospace Engineering, Cornell University Ithaca New York 14853 USA

## Abstract

The ability to change polymer properties has in the past largely been a factor of modulating the molecular weight, molecular weight distribution breadth, crosslinking, or branching. The use of controlled MWD shape has recently emerged as a promising avenue towards modifying polymer properties. Taking advantage of molecular weight distribution shape, we report a simple and efficient approach for tuning material properties in polystyrene-*block*-polyisoprene-*block*-polystyrene (SIS) thermoplastic elastomers (TPEs). We find that the skew of the MWD function governs tensile properties and can be used as a handle to predictably vary polymer toughness while reducing energy dissipation.

## Abstract 2

Taking advantage of molecular weight distributions shape, we report a simple and efficient approach for predictably tuning material properties for thermoplastic elastomers.

## Graphical abstract

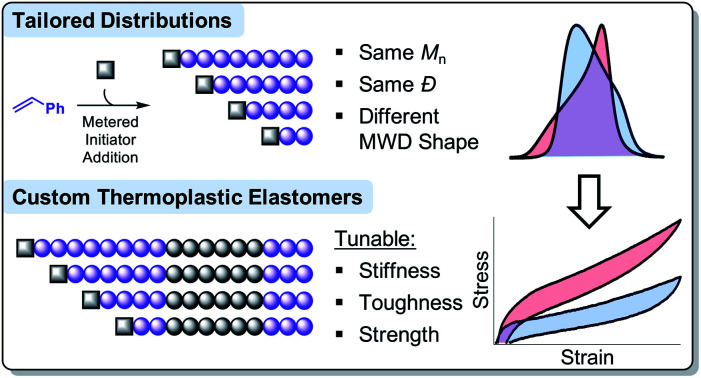

## Introduction

Structure–property relationships between polymer composition and tensile properties in thermoplastic elastomers (TPEs) have garnered significant interest for decades due to their wide use in a variety of applications including polymer modified asphalt, shoe soles, biomaterials, drug delivery, adhesives, and sealants.^1–7^ Commercial polystyrene-*block*-polyisoprene-*block*-polystyrene (SIS) TPEs, such as those produced by Kraton Polymers, contain narrow molecular weight distributions (MWDs) and achieve their elastomeric properties through a physically crosslinked network of hard polystyrene (PS) domains within a continuous rubbery polyisoprene (PI) matrix.^8^ While the presence of physical crosslinks allows for the material to be repeatedly reprocessed, such materials often experience high energy dissipation, or hysteresis energy, leading to undesired heat generation over time and ultimately premature failure.^9^ Therefore, a practical challenge has been to develop TPEs with increased resistance to high dissipation without compromising properties such as tensile strength and elasticity.

A persistent conception is that narrow MWDs, such as those found in many commercialized TPEs, are essential for formation of well-defined physical crosslinks and therefore high-performance properties. An early example supporting this notion was presented in a study by Morton and co-workers, who found that tensile strength in styrenic triblock copolymers decreased with an increase in dispersity (*Đ*) or breadth of the soft midblock.^10^ Interestingly, this study also demonstrated that aside from tensile strength, most other tensile properties were mainly dependent on the relative PS content rather than absolute block sizes. The impact of hard block content on material properties was also the focus of work by López-Barrón who found that hysteresis increased with a greater content of low molar mass PS homopolymer incorporated into SIS.^11^ Additionally, it has been suggested that broadening the dispersity of the hard block segments reduces storage modulus by disrupting domain perfection and by decreasing the fraction of chains with sufficiently high molar mass that contribute to physical crosslinks.^12,13^ Furthermore, there have been various reports on the influence of block length and *Đ* on polymer microphase behaviour, which has a direct influence on bulk properties.^14–19^ While these reports provide important information regarding the influence of block size and midblock MWD breadth on elastomeric properties, there remains an opportunity to use the entire endblock MWD shape as a means to fine-tune TPE properties.

**Fig. 1:**
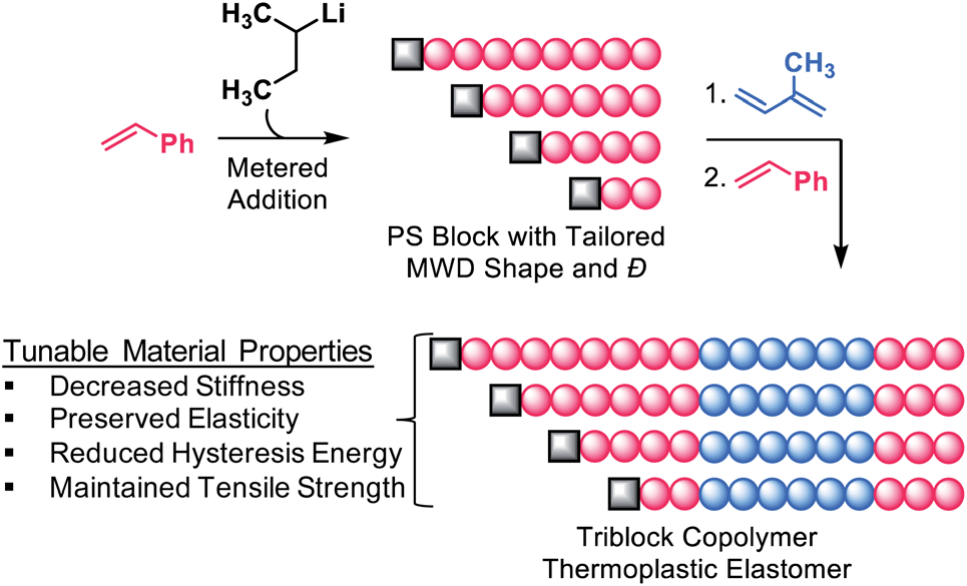
General strategy for the preparation of SIS triblock copolymers in which the MWD of the first PS block is skewed. The breadth of the distribution is controlled by the rate of addition, such that *Đ* broadens with an increase in addition time. Refer to Fig. S2† for details.

Recently, new synthetic methods have emerged that provide control over MWD shape.^16–18,20–25^ In particular, our group has developed a versatile strategy facilitating absolute control of MWD shape through temporal regulation of polymer chain initiation in both controlled radical and anionic polymerisations.^16–18,20–23^ Using this method, we showed that the Young's modulus of PS-*b*-PI copolymers could be varied up to 3.5-fold by altering the skew of the PS MWD.^21^ We have also used our synthetic approach to elucidate the impact of MWD shape on diblock copolymer self-assembly.^16,17^ These results clearly demonstrated that MWD shape is just as important as MWD breadth in determining polymer physical properties. With these results in mind, we sought to explore the influence of MWD shape on SIS triblock copolymer TPEs. We envisioned that manipulation of the shape of the PS endblock would serve as a platform for tailoring the properties of commercial TPEs. Herein, we prepared a library of SIS triblock copolymer TPEs in which the MWD shape and *Đ* of the first PS endblock was systematically varied (Fig. 1), and determined the tensile characteristics of the materials. We found that the shape of the MWD, rather than the breadth alone, governed polymer stiffness, tensile strength, and energy storage/dissipation properties. Our results demonstrate that MWD shape can be strategically used to produce TPEs with finely tuned material properties and reduced hysteresis energy. This study explicitly investigates the influence of well-defined MWD shapes on the tensile properties of TPEs.

## Experimental

### Materials

All reactions were performed in a Unilab MBraun Glovebox with a nitrogen atmosphere. *sec*-Butyllithium (*sec*-BuLi, Sigma Aldrich, 1.4 M in cyclohexane), butylhydroxytoluene (BHT) (TCI, >99.0%), chloroform-D (CDCl_3_, Cambridge Isotope Laboratories Inc., 99.8%), methanol (MeOH, 99.8%, Fisher Scientific), and dichloromethane (DCM, 99.5%, Fisher Scientific) were used without further purification. Styrene (Sigma Aldrich, 99+%), isoprene (Sigma Aldrich, 99+%), and diphenylethylene (DPE, Sigma Aldrich, 97%) were dried over calcium hydride (CaH_2_) (ACROS organics, 93% extra pure, 0–2 mm grain size) for a minimum of 24 h. Styrene and DPE were vacuum transferred and degassed by three freeze–pump–thaw cycles. Isoprene was vacuum transferred onto activated 4 Å molecular sieves (EMD Chemicals, 8–12 mesh beads) for further drying and after 48 hours was vacuum transferred and degassed by three freeze–pump–thaw cycles. Molecular sieves were activated under vacuum at 180 °C overnight. Cyclohexane was degassed by two freeze–pump–thaw cycles before a 1 : 1.2 molar ratio of DPE and *sec*-BuLi was added under a nitrogen blanket until a deep red colour was sustained. This solution was stirred for a minimum of 1 hour. Cyclohexane was distilled under nitrogen from the DPE/*sec*-BuLi and additionally degassed by three freeze–pump–thaw cycles.

### Analytical methods

All polymer samples were analysed using a Tosoh EcoSec HLC 8320GPC system with two SuperHM-M columns in series at a flow rate of 0.350 mL min^−1^. THF was used as the eluent and all number-average molecular weights (*M*_n_), weight-average molecular weights (*M*_w_), dispersities (*Đ*), asymmetry factors (*A*_s_), *M*_*z*_ and *M*_*z*+1_ for the first polystyrene block were calculated from refractive index chromatograms against TSKgel polystyrene standards. Conversions were determined by ^1^H nuclear magnetic resonance (NMR) spectra obtained on a Bruker 500 MHz NMR spectrometer in CDCl_3_, as were the *M*_n_s for the diblock and triblock copolymers.

### Synthesis

#### Synthesis of SIS copolymer with narrow MWDs

A 20 mL scintillation vial equipped with a magnetic stirrer was flame dried, brought into the glovebox, and charged with 14 mL of cyclohexane and 275 μL of styrene (2.4 mmol). A *sec*-BuLi stock solution in cyclohexane (0.1 M) was prepared for the reactions. The stir plate was set to 600 rpm, and 200 μL of *sec*-BuLi (0.02 mmol) was quickly added in one portion, giving a yellow solution indicating formation of the polystyryl anion. The reaction was capped and stirred for approximately 4 h, allowing for full monomer conversion. Then, 2.3 mL of isoprene (23 mmol) was added and the reaction colour quickly faded from yellow to clear, indicative of the polyisoprenyl anion. The stir plate was adjusted to 1000 rpm to account for the increased viscosity of the polyisoprenyl anion. After 12 h, 275 μL styrene (2.4 mmol) was added and the reaction vial was placed in a heating block equipped with a thermocouple. The reaction was heated to 40 °C and allowed to stir for 5 h. The colour of the reaction slowly changed from clear to yellow. The polymerization was quenched with addition of BHT and vigorously shaken until the reaction colour completely faded. The reaction vial was removed from the glovebox and the polymer was precipitated once from MeOH. The polymer was dissolved in DCM and 0.4 mL of BHT in DCM (10 mg BHT/1 mL DCM) was added as a stabilizer. The polymer solution was concentrated *via* rotary evaporation and polymers were dried in a vacuum oven at 60 °C for 12 h.

#### Synthesis of SIS copolymer with a skewed MWD

A 20 mL scintillation vial equipped with a magnetic stirrer was flame dried, brought into the glovebox, and charged with 2 mL of cyclohexane and 275 μL of styrene (2.4 mmol). A *sec*-BuLi stock solution in cyclohexane (0.033 M) was prepared for the reactions and a total volume of 650 μL of the solution was drawn into a 1 mL syringe and then mounted onto a New Era NE-4000 Double Syringe Pump. The pump was programmed according to the appropriate rate profile (Tables S1 and S2†), which would dispense a total volume of 615.3 μL (0.02 mmol) of the initiator solution. Once the needle was submerged into the reaction mixture, the stir plate was set to 350 rpm and the addition program was started. At full addition of *sec*-BuLi, the reaction was capped and stirred at 500 rpm until full conversion of styrene to polystyrene was reached. 12 mL of cyclohexane followed by 2.3 mL of isoprene (23 mmol) were next added. The reaction colour quickly faded from yellow to clear after addition of isoprene. The stir plate was adjusted to 1000 rpm to account for the increased viscosity of the polyisoprenyl anion. After 12 h, 275 μL styrene (2.4 mmol) was added and the reaction vial was placed in a heating block equipped with a thermocouple. The reaction was heated to 40 °C and allowed to stir for 5 h. The colour of the reaction slowly changed from clear to yellow. The polymerization was quenched with addition of BHT and vigorously shaken until the colour of the reaction completely faded. The reaction vial was removed from the glovebox and the polymer was precipitated once from MeOH. The polymer was dissolved in DCM and BHT (0.4 mL of 10 mg BHT/1 mL DCM) was added as a stabilizer. The polymer solution was concentrated *via* rotary evaporation. Polymers were dried in a vacuum oven at 60 °C for 12 h.

### Material testing

#### Sample preparation for cyclic testing

Compression moulding was carried out using a 4120 Hydraulic Unit Carver press, a stainless-steel dog bone mould (see Fig. S9†), and PTFE protective sheets (CS Hyde). The dog bone mould was custom ordered from the Laboratory of Atomic and Solid State Physics Machine Shop at Cornell University. The mould was sprayed with PTFE (Sprayon, MR 311) to prevent polymer from adhering to the mould. Specimens were prepared by compression moulding for 1 minute between PTFE protective sheets at 130 °C under 3000 lbs of pressure. Samples were cooled to 25 °C by cooling the plates with a stream of water, and excess polymer was trimmed from the specimens. Final dog bone specimens had uniform dimensions with a cross sectional area of 1.86 mm^2^.

#### Tensile testing

Tensile properties of compression-moulded copolymer samples were analysed using a Zwick/Roell Z010 testing system equipped with pneumatic grips and analysed using Zwick/Roell TestXpert II v.3.5 software. Dog bone specimens were clamped using pneumatic grips pressurized to 120 psi. Samples were loaded to either 100, 300, or 500 percent strain, followed immediately by unloading to the original length. For both loading and unloading, a strain rate of 0.01 s^−1^ was used. Toughness (*U*_T_) was calculated as the area under the loading curve, while hysteresis energy (*W*_H_) was calculated as the area between the loading and unloading curves. Areas were calculated using the trapezoidal method. Young's modulus (*E*) was determined from the loading curve as the slope of the linear elastic region at low (<10%) strain.

## Results and discussion

**Fig. 2:**
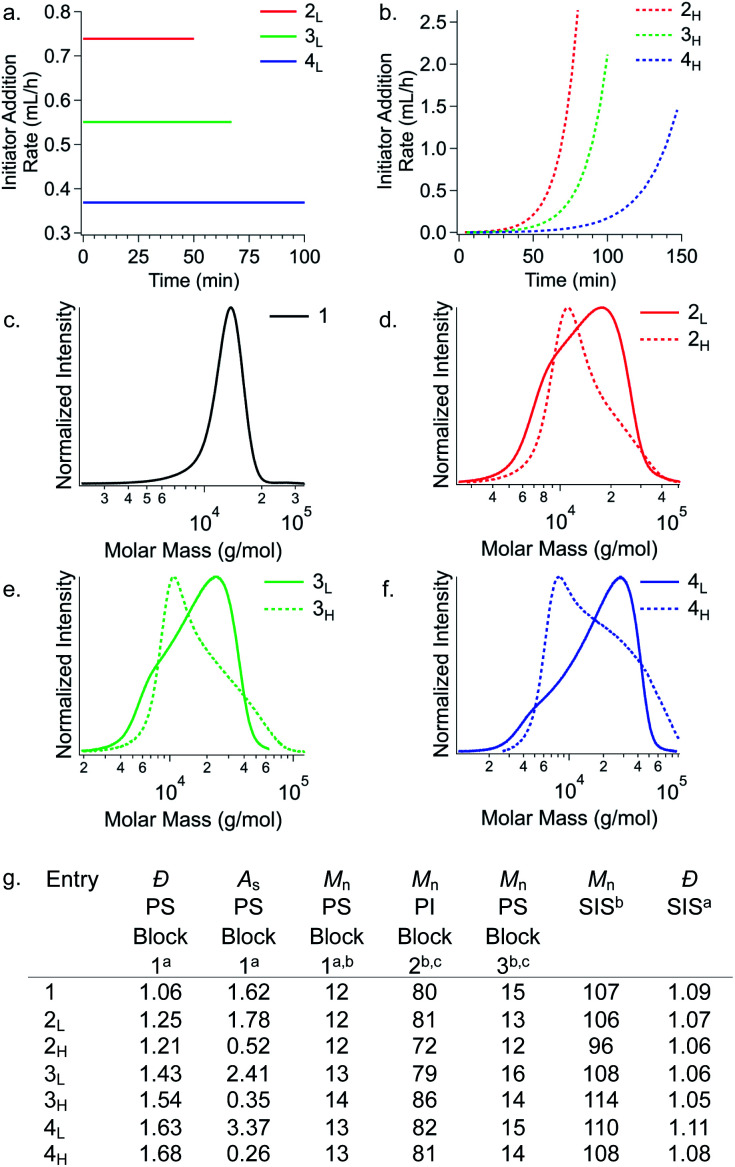
Rate addition profiles for PS initiated either by (a) a constant rate of *sec*-BuLi addition or by (b) an exponentially increasing rate of *sec*-BuLi addition. (c–f) Molecular weight distributions of the first PS block highlighting the differences in shape for polymers with the same *Đ*. (g) Chain extension of various shape/*Đ* controlled PS blocks provides SIS copolymers differing only in their MWD skew of the first PS block. ^*a*^Determined from RI SEC traces. ^*b*^*M*_n_s are given in kg mol^−1^. ^*c*^Determined by ^1^H NMR spectra. For SEC traces showing chain extension, see Fig. S3.†

Temporal control of initiation (Fig. 1) enabled us to dictate the shape and breadth of the first PS block (*Đ*_PS_) by metered addition of *sec*-BuLi to a polymerisation reaction of styrene, affording polymers with precisely tailored MWD compositions (Fig. 2a–d). Living PS blocks with tailored MWDs were subsequently chain extended with isoprene and styrene, providing SIS copolymers with number-average molecular weights (*M*_n_s) ranging between 96–114 kg mol^−1^ with overall *Đ* ∼ 1.1 (Fig. 2e). Polymer **1**, composed entirely of blocks with narrow distributions, was prepared as a reference. Polymers **2L** and **2H** were prepared as a complementary set, both having an outer PS block with *Đ* ∼ 1.23, but with opposite MWD shapes tailed to lower or higher MW, respectively. Polymers **3L** and **3H** also have oppositely skewed PS first blocks with a *Đ*_PS_ ∼ 1.49. Polymers **4L** and **4H** have complementary MWDs with a *Đ*_PS_ ∼ 1.66.

**Fig. 3:**
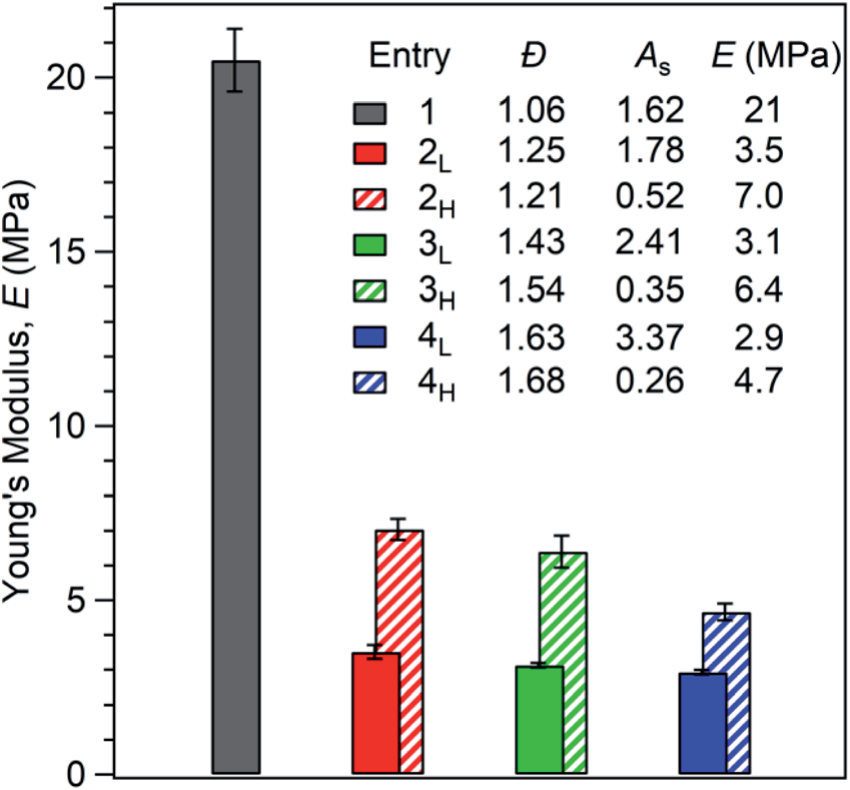
Effect of first PS block *Đ* and shape on Young's modulus (*E*). Each value of *E* is an average of at least three measurements.

To initiate materials characterisation, we began by compression moulding the polymers into dog bone specimens and subjecting them to tensile testing. First, we considered each polymer's Young's modulus (*E*). We observed that increasing *Đ*_PS_ reduces stiffness. Additionally, polymers with high MW tails have a greater *E* than polymers with low MW tails (Fig. 3). Specifically, the Young's modulus of the reference polymer **1** (*E* = 21 MPa) is higher than all polymers with broader PS MWDs. Interestingly, samples **2L** and **2H** (*E* = 3.5 MPa and 7.0 MPa, respectively) show a 67% difference in *E* at the same MWD breadth (*Đ*_PS_ ∼ 1.23). Polymers **3L** and **3H**, which both have *Đ*_PS_ ∼ 1.49 exhibit similar differences in *E* of 69% (*E* = 3.1 MPa and 6.4 MPa, respectively). At *Đ*_PS_ ∼ 1.66, polymers **4L** and **4H** differ by 46% (*E* = 2.9 MPa and 4.7 MPa, respectively). These results demonstrate that both MWD *Đ* and shape influence *E*. Significantly, polymers with MWDs tailing towards higher molar mass have substantially higher stiffness than their MWD shape counterparts at the same value of dispersity. These results are consistent with our previous observations on PS-*b*-PI diblock copolymers.^21^

**Fig. 4:**
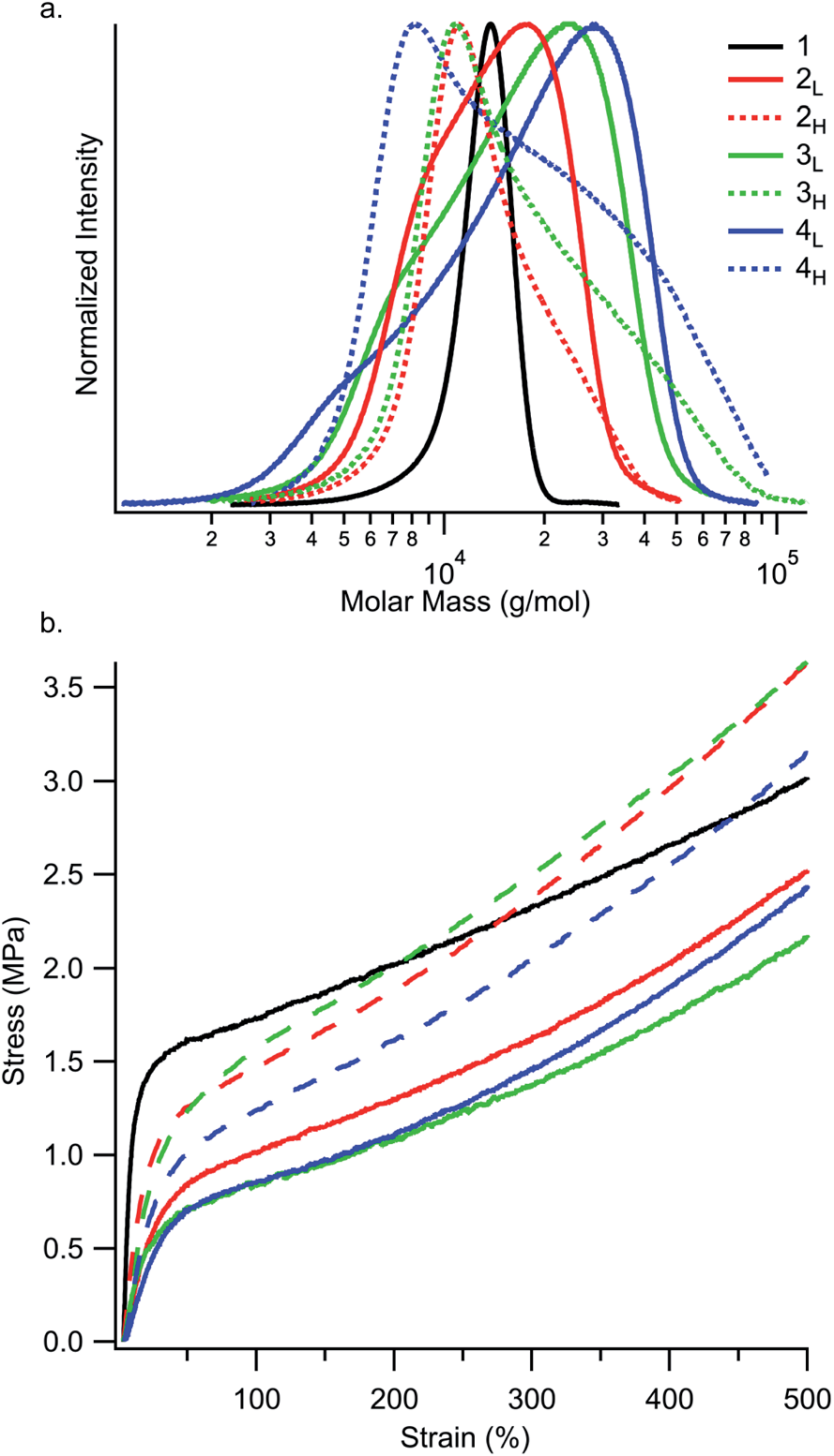
(a) Molecular weight distributions of the first PS block in the SIS thermoplastic elastomers. The *y*-axis intensities have been normalized to highlight the differences in MWD shape. (b) Stress–strain curves display a region of linear elastic behaviour at low strain values. The end of the linear elastic region is marked by yielding, followed by strain hardening. Each displayed stress–strain curve is an average of three specimens. See Tables S3 and S4† for yielding and strain hardening rate values.

Next, we examined the yielding behaviour of these materials (Fig. 4). We hypothesized that changing the skew of the PS block would change the point at which hard PS domains rupture into smaller domains.^26^ We observed the highest yield stress in the reference polymer **1**, and that yield stress generally decreases with increasing *Đ*_PS_ (Table S3†). Moreover, the lowest yield stresses were found in polymers **2L**, **3L**, and **4L**, which have tailing towards low MW. We attribute this relationship to the fact that PS domains containing a broad distribution of chains have a higher fraction of low MW chains. Resultantly, these polymers may experience more chain pullout from PS domains, as relaxations in diblock and triblock copolymers are biased towards lower molar mass species.^27^ This behaviour leads to a higher yield stress for polymers with narrower dispersity, and, more interestingly, to a lower yield stress in polymers with MWD tailing towards low molar mass compared to the samples with the same *Đ* value but MWD tailing towards high molar mass.

In addition to yielding, we explored the tensile strength and strain hardening of this set of SIS copolymers. It has been well studied that chain conformation of the middle block in ABA triblock copolymers is influential on material properties, including strength and strain hardening.^28–31^ The midblock can attain two conformations: a loop, in which both hard block chain ends are within the same A domain, or a bridge, in which chain ends are anchored within two different A domains. Bridging chains greatly enhance polymer strength by linking glassy PS domains, whereas loop chains behave more similarly to AB diblock copolymers.^26^ Following yielding and pullout of very short chains, the effective chain length composition held within the PS domains shifts to higher MW. These higher molar mass species should have a much higher energy barrier to pullout.^27^ As such, we propose that the presence of high MW chains in the PS MWD would reinforce bridges by resisting chain pullout from PS domains. This resistance to chain pullout would result in an increase in strain hardening rate, seen as a steeper slope in the stress strain curve following yielding.

While the reference polymer **1** has PS chains only within a narrow range around 12–14 kg mol^−1^, our disperse samples have significant fractions of chains well above 20 kg mol^−1^ (Fig. 4a). As seen in Fig. 4b and Table S4,† strain hardening rate is highest in polymers with MWDs tailing towards high molar mass (**2H**, **3H**, and **4H**) and lowest in the reference polymer **1**. An interesting consequence of the increased strain hardening rate experienced by polymers with broadened *Đ*_PS_ is that their tensile strengths at 500% elongation are still competitive with or better than the reference polymer **1** (3.0 MPa), despite their diminished yield stresses. At 500% elongation, high MW skewed polymers **2H** (3.6 MPa), **3H** (3.6 MPa), and **4H** (3.2 MPa) have higher tensile strengths than low MW skewed polymers **2L** (2.5 MPa), **3L** (2.2 MPa), and **4L** (2.5 MPa). This highlights the dependence of tensile strength on MWD shape. Motivated by these results, we then went on to investigate the influence of MWD shape on deformation mechanisms and energy dissipation properties.

Deformation mechanisms in SIS under tensile load have been studied and are well understood.^11,26,28,32–36^ Initially in the linear elastic region, SIS deforms in a fully reversible manner. This reversible behaviour is then disrupted by micro-yielding events which lead to full yielding of the hard PS domains. Following yielding, PI chain stretching may occur, producing a plateau in the stress–strain curve. Disrupted PS domains then undergo various rearrangements until a new orientation is reached. Additional extension of SIS in its new orientation leads to further chain stretching and eventual rupture of the domains and the material. As these events occur at different stages along the stress–strain profile, it is possible to isolate them and study their response to varied MWD features. A simple way to do so is by measuring the hysteretic behaviour, or energy loss, of the materials, as such behaviour is related to deformation mechanisms.

**Fig. 5:**
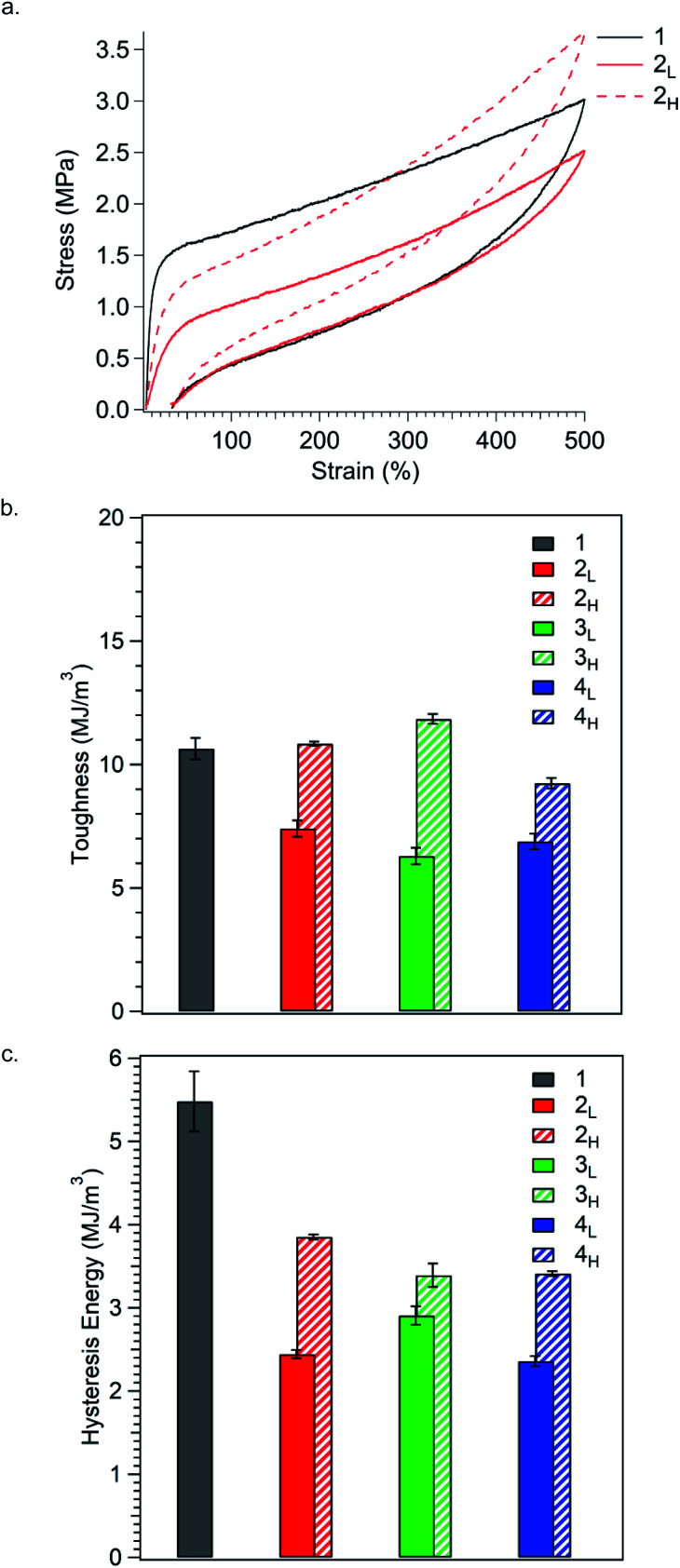
(a) Representative stress–strain curves for the load/unload cycle for polymers stretched to 500% elongations. Results are summarised as bar graphs showing (b) toughness and (c) hysteresis energy for the load/unload cycle. All values are an average of at least three measurements. Stress–strain curves and results from stretching all samples to 100%, 300%, or 500% elongation can be found in Fig. S4–S8.† See Table S5† for values.

TPEs typically exhibit significant hysteresis, occurring mainly through viscous flow, microstructural breakdown, and subsequent irreversible rearrangement of the macromolecular chain network in the hard domains.^26^ In order to measure the hysteretic response of the materials, we conducted a series of loading–unloading experiments in which polymers were stretched to either 100, 300, or 500% elongation followed by compression to zero force (Fig. S4–S6,† and 5a). Toughness (*U*_T_) was taken as the total area underneath the loading curve (Fig. 5b), while hysteresis energy was calculated as the area between the loading and unloading curves (Fig. 5c).

Polymer toughness, much like tensile strength, increases in TPEs as crosslinks are reinforced.^37^ As such, the inclusion of high MW species in the glassy domains should increase toughness by resisting chain pullout, thereby enhancing the strength of bridging chains. Conversely, incorporating low MW chains should decrease toughness, as these chains are less resistant to chain pullout. Expectantly, we found that polymers with PS MWDs tailing towards higher MW were much tougher than polymers with MWDs tailing towards low MW (Fig. 5b). At 500% elongation, the reference polymer **1** (*U*_T_ = 11 MJ m^−3^) is similar in toughness to polymers **2H**, **3H**, and **4H**, all having PS MWDs tailing towards higher molar mass (*U*_T_ = 11 MJ m^−3^, 12 MJ m^−3^, and 9.3 MJ m^−3^, respectively). In contrast, polymers **2L**, **3L**, and **4L**, have PS MWDs tailing towards lower molar mass and have significantly reduced toughness (*U*_T_ = 7.4 MJ m^−3^, 6.3 MJ m^−3^, and 6.9 MJ m^−3^, respectively). These results corroborate our hypothesis that MWD shape impacts bridge chain strength, specifically, that high MW chains positively impact bridge chains strength and low MW chains compromise such strength. Moreover, polymers with the same MWD shape but different dispersities are similarly tough, suggesting that MWD shape has profound influence on toughness. The trend in toughness observed at 500% elongation is also observed at 100% and 300% strain, indicating that the dependence of toughness on polymer composition remains constant throughout the entire stress/strain curve (Fig. S7a and S8a†).

Perhaps most striking is that while MWD shape can be used to increase or decrease toughness relative to the reference polymer, hysteresis energy is significantly reduced for both MWD shapes (Fig. 5c). At 500% elongation, the reference polymer **1** has the highest hysteresis energy (5.5 MJ m^−3^), and simply by controlling the *Đ*_PS_, hysteresis energy can be reduced by up to 80% (entry **4L**). Additionally, changing the shape of the MWD offers the ability to fine tune hysteresis energy. Polymers with high MW tails in their MWDs (**2H**, **3H**, and **4H**) have higher values for hysteresis energy (3.9 MJ m^−3^, 3.5 MJ m^−3^, and 3.4 MJ m^−3^, respectively) compared to polymers **2L** (2.4 MJ m^−3^), **3L** (2.9 MJ m^−3^), and **4L** (2.4 MJ m^−3^), each having low MW tails. We believe this dependence on MWD shape to be an effect of chain pullout at high extension. As more chains are pulled away from PS domains, the fraction of total PS chains contributing to the physically crosslinked network decreases, which reduces friction between polymer chains within PS domains. A reduction in internal chain–chain friction corresponds to a reduction in hysteresis energy.^35,38,39^ Expectantly, polymers with MWDs tailing towards low molar mass have the lowest values of hysteresis energy, since lower MW chains can be pulled away from PS domains more easily than higher MW chains. Using this internal friction based argument, the overall reduction in hysteresis energy for polymers with increased *Đ*_PS_ relative to the reference polymer is then unsurprising, as polydisperse systems have already been shown to possess decreased internal friction relative to monodisperse systems.^40–44^

## Conclusions

Our results show that deliberate modification of MWDs affords polymers with the same block lengths but with considerably different material properties. We suspect that the entire MWD breadth and shape is responsible for varied properties. Specifically, increasing *Đ*_PS_ enhances initial flexibility by reducing Young's modulus and yield strength. Beyond yielding, increasing *Đ*_PS_ was found to increase strain hardening. Moreover, the skew of the MWD has important implications to material properties. We propose that increasing the portion of low MW PS chains leads to significant chain pullout from PS domains at relatively low strain, thus decreasing polymer stiffness as well as yield stress. Furthermore, by increasing the portion of high molar mass PS chains, we observed an increase in strength, strain hardening, and toughness, which we attribute to a reinforcement of bridging chains. These results demonstrate that control over MWD shape, or more specifically chain composition, facilitates the production of polymers with precisely tuned material properties. For applications in which high stiffness and yield strength are prioritized, the reference polymer **1** is perhaps most suitable. However, for applications in which failure due to heat generation is of particular concern, our polymers with broadened and skewed MWDs are advantageous. Particularly, polymer **3H** combines exceptional toughness with low hysteresis energy while maintaining excellent elasticity and tensile strength at 500% elongation. Further studies will investigate the role of varied MWD shapes of multiple blocks in SIS copolymers on their material properties.

## Conflicts of interest

There are no conflicts to declare.

## Supplementary Material

SC-011-C9SC05278J-s001
