# Supplementary material for: Tailor-made thermoplastic elastomers: customisable materials via modulation of molecular weight distributions
Source: Chem Sci. 2019 Dec 17;11(5):1361–7. doi: 10.1039/c9sc05278j (PMC8148047; doi:10.1039/c9sc05278j)
Supplement: SC-011-C9SC05278J-s001 [file SC-011-C9SC05278J-s001.pdf]

Electronic Supplementary Material (ESI) for Chemical Science.
This journal is © The Royal Society of Chemistry 2019

Supporting Information

Tailor-Made Thermoplastic Elastomers: Customizable Materials via Modulation of Molecular Weight Distributions

Stephanie I. Rosenbloom[†] Dillon T. Gentekos[†] Meredith N. Silberstein,[‡] and Brett P. Fors[†]

[†]Department of Chemistry and Chemical Biology, Cornell University, Ithaca, New York, 14853, United States

[‡]Sibley School of Mechanical and Aerospace Engineering, Cornell University, Ithaca, New York 14853, United States

Table of Contents

General procedure for Dynamic Mechanical Analysis (DMA)……………………………..………S2
General procedure for Small-Angle X-ray Scattering (SAXS)……………………………….…… S2
Fig. S1. Illustration for the calculation of the asymmetry factor (A_s)………………………………..S2
Fig. S2. Controlling the shape and breadth of the MWD of the first PS block……………………....S3
Fig. S3. Library of SIS triblock copolymers prepared……………………………………………….S3
Procedure for Fig. S3: Constant Rate Addition Profiles…………………………….………...S4
Table S1. Constant Rate Addition Profiles………………………………………………………..S4
Procedure for Fig. S3: Exponentially Ramped Addition Rate Profiles……………………….…….S4
Table S2. Exponentially Ramped Rate Addition Profiles……………………………………………S4
Table S3. Average Yield Stress and Yield Strain…………………………………………………….S5
Table S4. Average Strain Hardening Rates……………...……………………………………..S5
Fig. S4. Stress-strain curves showing loading/unloading cycle to 100% elongation……………………S6
Fig. S5. Stress-strain curves showing loading/unloading cycle to 300% elongation…………………....S6
Fig. S6. Stress-strain curves showing loading/unloading cycle to 500% elongation…………………....S7
Fig. S7. Bar graph results from loading/unloading cycle to 100% elongation…………………………S7
Fig. S8. Bar graph results from loading/unloading cycle to 300% elongation……………………… ...S8
Table S5. Average hysteresis energy and toughness for loading/unloading cycles……………………..S8
Fig. S9. Specifications for stainless steel dog bone mould……………………………………….….S9
Fig. S10. Representative ^{1}H NMR of SIS triblock copolymer……………………………………....S9
Fig. S11. SAXS traces of SIS samples with similar polymer composition…………………………….S10
Table S6. Stress Relaxation and Strain Recovery from Dynamic Mechanical Analysis………………S11
Fig. S12. Stress/strain curves of polymer 1 in pristine and reprocessed conditions……………………S11
References ……………………………………………...……………………………………….S11

General procedure for Dynamic Mechanical Analysis (DMA). Polymers were compression moulded under 3,000 lbs of pressure for 1 minute at 130 °C into straight specimens. Stress relaxation and strain recovery data were obtained on a TA Instruments DMA Q800 fitted with tension grips. Samples were stretched to 5% strain and held there to afford a total stress relaxation time of 10 minutes. Strain recovery was measured for 10 minutes after load removal. Each stress relaxation and strain recovery value are an average of three measurements.

General procedure for Small-Angle X-ray Scattering (SAXS). SIS samples were prepared by solution casting polymer samples (6 mL, 7 wt% in toluene) into aluminum pans and allowing the solvent to slowly evaporate. The samples were subsequently thermally annealed at 130 °C in a vacuum oven for 48 h before cooling to room temperature under vacuum. After annealing, polymers were removed from aluminum pans, and a small piece was cut from each polymer to be placed into the center of a stainless steel washer (4.42 mm I.D., 9.53 mm O.D., 0.79 mm thickness). The washers were sealed between Kapton tape, and were used directly for the small-angle X-ray scattering measurements which were performed at the G1 beamline at Cornell High Energy Synchrotron Source (CHESS). 2D-SAXS patterns were recorded with a Dectris Eiger 1M detector (1030 x 1065 pixels, 77 x 80 mm2 active area) at a sample to detector distance of 2.027 m and an X-ray wavelength (λ) of 1.252 Å. The data was corrected for detector response, calibrated with silver behenate, and, using the Nika package in Igor Pro,[1] reduced by azimuthally integrating the 2D pattern to acquire a 1D plot of intensity versus the scattering wavevector (q).

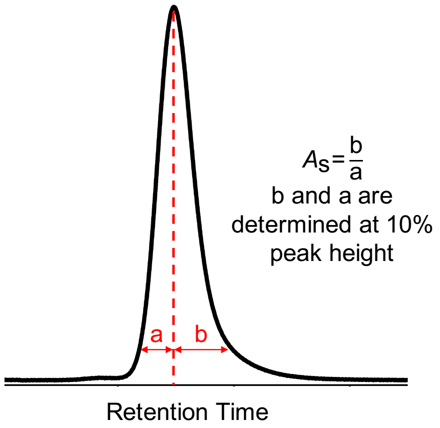

Fig. S1. Illustration for the calculation of the asymmetry factor (A_s).[2]

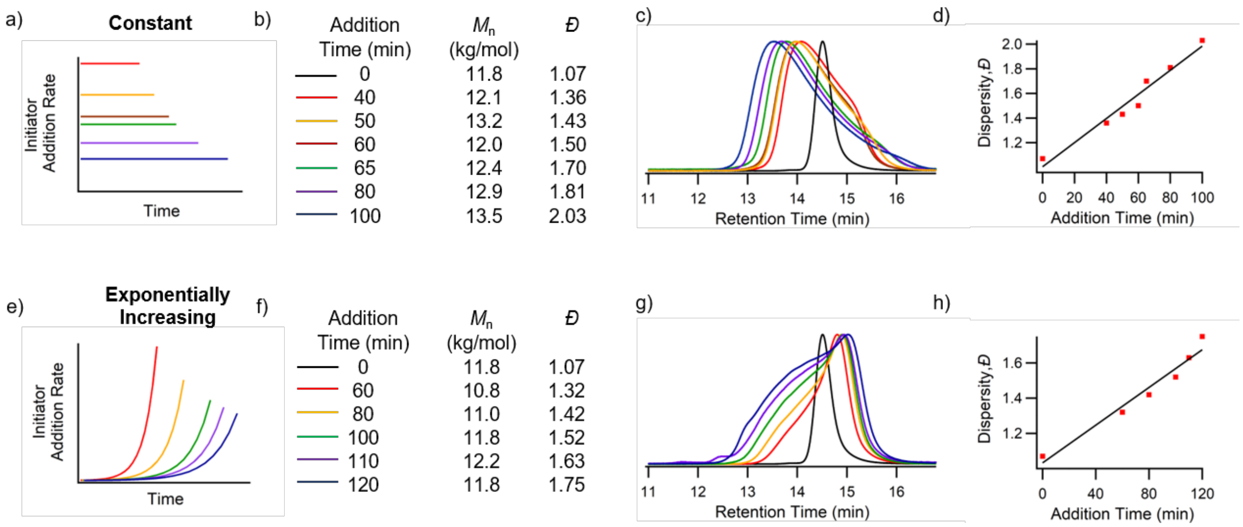

Addition Time (min)	M_n (kg/mol)	$Đ$
0	11.8	1.07
40	12.1	1.36
50	13.2	1.43
60	12.0	1.50
65	12.4	1.70
80	12.9	1.81
100	13.5	2.03

Addition Time (min)	M_n (kg/mol)	$Đ$
0	11.8	1.07
60	10.8	1.32
80	11.0	1.42
100	11.8	1.52
110	12.2	1.63
120	11.8	1.75

Fig. S2. Controlling the shape and breadth (dispersity, $Đ$) of the MWD of the first PS block in PS-*b*-PI-*b*-PS (SIS) triblock copolymer with constant (a-d) and exponentially ramped (e-h) rates of initiator addition.

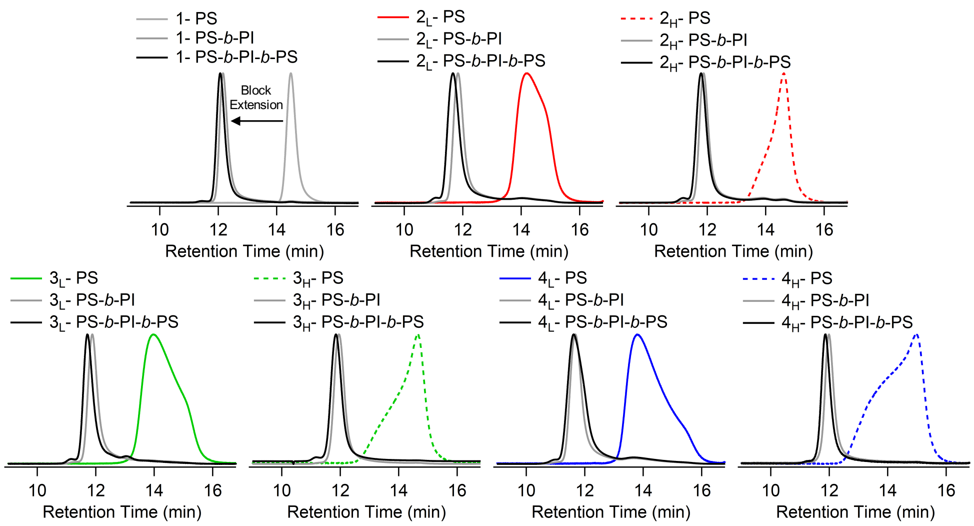

Entry	$Đ$ PS Block 1[a]	A_s PS Block 1[a]	M_n PS Block 1[a,b]	M_n PI Block 2[b,c]	M_n PS Block 3[b,c]	M_n SIS[b]	$Đ$ SIS[a]
1	1.06	1.62	12	80	15	107	1.09
2_L	1.25	1.78	12	81	13	106	1.07
2_H	1.21	0.52	12	72	12	96	1.06
3_L	1.43	2.41	13	79	16	108	1.06
3_H	1.54	0.35	14	86	14	114	1.05
4_L	1.63	3.37	13	82	15	110	1.11
4_H	1.68	0.26	13	81	14	108	1.08

Fig. S3. Library of SIS triblock copolymers prepared via chain extension from PS with varying degrees of skewness and $Đ$. [a]Determined from RI GPC traces. [b]M_ns are given in kg/mol. [c]Determined by ^1H NMR spectra. Subscripts "L" and "H" indicate the direction of tailing in the first PS block MWD, either towards low or high molar mass species.

Procedure for Fig. S3: Constant Rate Addition Profiles

The synthesis was performed according to the general procedure. The New Era NE4000 Double Syringe Pump was programmed according to Table S1.

Table S1. Constant Rate Addition Profiles

Addition Time (min)	Addition Rate (µL/h)	Total Volume (µL)
50	739	615
67	551	615
100	369	615

Procedure for Fig. S3: Exponentially Ramped Addition Rate Profiles

The synthesis was performed according to the general procedure. All exponentially increasing addition profiles were programmed as a sequence of 20 step increments with each step corresponding to a phase in the New Era NE4000 Double Syringe Pump program, according to Table S2.

Table S2. Exponentially Ramped Rate Addition Profiles

Step #	Rate (µL/h)			Volume /Step (µL)
	80 min	100 min	147 min	
**1**	4.4	3.5	2.4	0.3
**2**	6.2	4.9	3.4	0.4
**3**	8.7	6.9	4.8	0.6
**4**	12	9.7	6.7	0.8
**5**	17	14	9.4	1.1
**6**	24	19	13	1.6
**7**	33	27	18	2.2
**8**	47	37	26	3.1
**9**	65	52	36	4.3
**10**	91	73	50	6.1
**11**	128	102	71	8.5
**12**	179	143	99	12
**13**	251	200	138	17
**14**	351	281	194	23
**15**	491	393	271	33
**16**	687	550	379	46
**17**	962	770	531	64
**18**	1347	1078	743	90
**19**	1886	1509	1041	126
**20**	2641	2112	1457	176

Table S3. Average Yield Stress and Yield Strain

Entry	Đ PS Block 1	A_s PS Block 1	Yield Stress (MPa)	Yield Strain (%)
1	1.06	1.62	1.5	27
2_L	1.25	1.78	0.8	44
2_H	1.21	0.52	1.2	36
3_L	1.43	2.41	0.7	38
3_H	1.54	0.35	1.1	38
4_L	1.63	3.37	0.7	48
4_H	1.68	0.26	0.9	42

Table S4. Average Strain Hardening Rates

Entry	Đ PS Block 1	A_s PS Block 1	Strain Hardening 1 (MPa/ε)	Strain Hardening 2 (MPa/ε)
1	1.06	1.62	0.3	0.3
2_L	1.25	1.78	0.3	0.5
2_H	1.21	0.52	0.5	0.6
3_L	1.43	2.41	0.3	0.4
3_H	1.54	0.35	0.5	0.6
4_L	1.63	3.37	0.3	0.5
4_H	1.68	0.26	0.4	0.6

Due to the nonlinearity of the stress/strain curves following yielding, stress/strain curves were divided into two regions each with linear slopes. Strain hardening 1 is the slope measured from the yield strain to a strain (ε) of three (corresponding to 300% elongation). Strain hardening 2 is the slope measured from $\varepsilon = 3$ to $\varepsilon = 5$ (corresponding to 300-500% elongation).

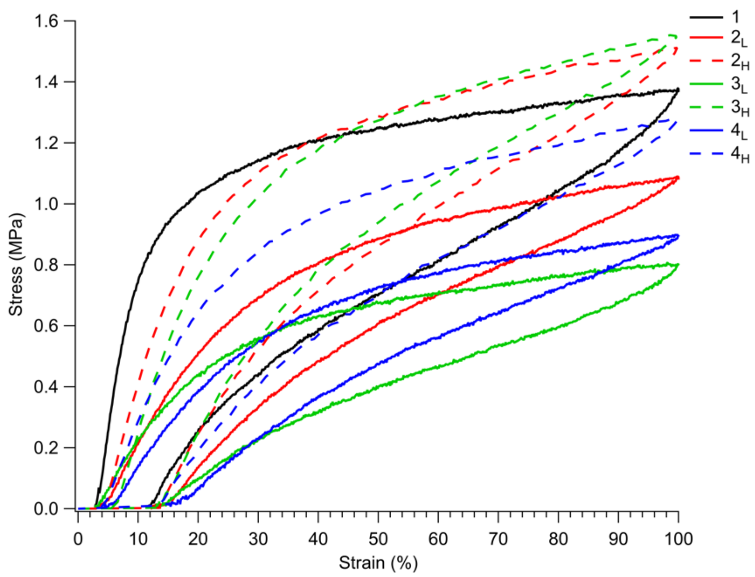

Fig. S4. Stress-strain curves showing loading/unloading cycle for polymers stretched to 100% elongation. Each displayed stress-strain curve is an average of three specimens.

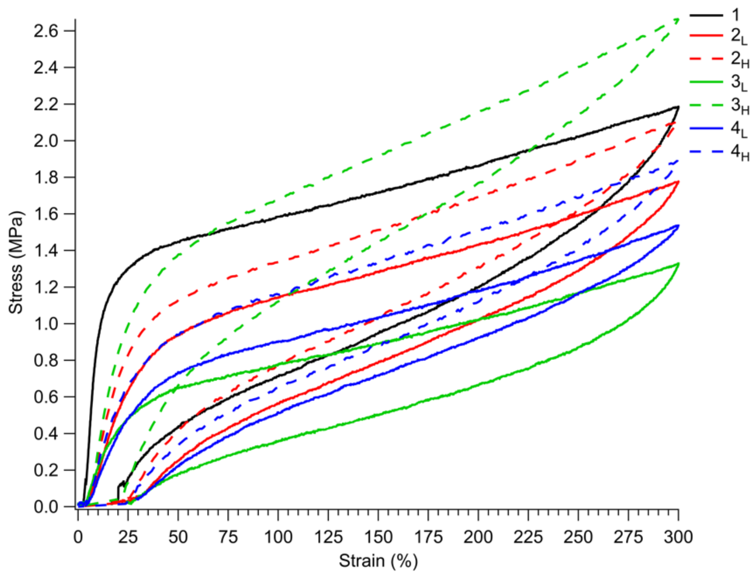

Fig. S5. Stress-strain curves showing loading/unloading cycle for polymers stretched to 300% elongation. Each displayed stress-strain curve is an average of three specimens.

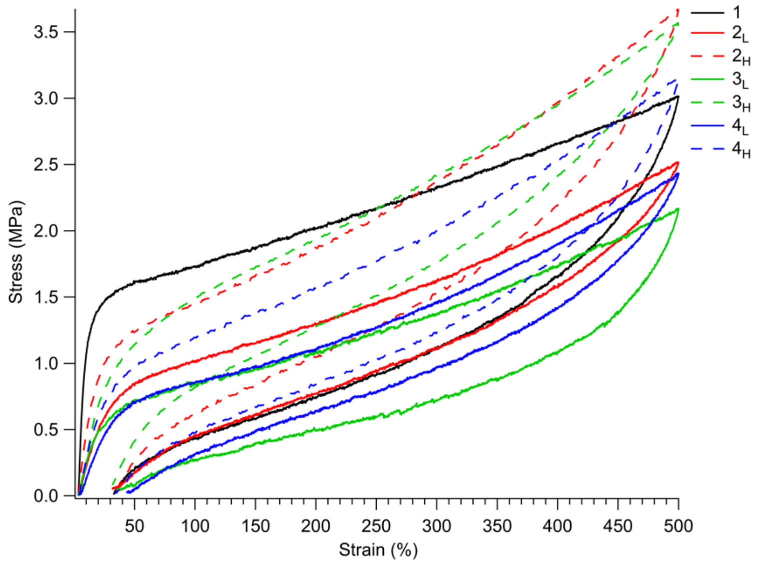

Fig. S6. Stress-strain curves showing loading/unloading cycle for polymers stretched to 500% elongation. Each displayed stress-strain curve is an average of three specimens.

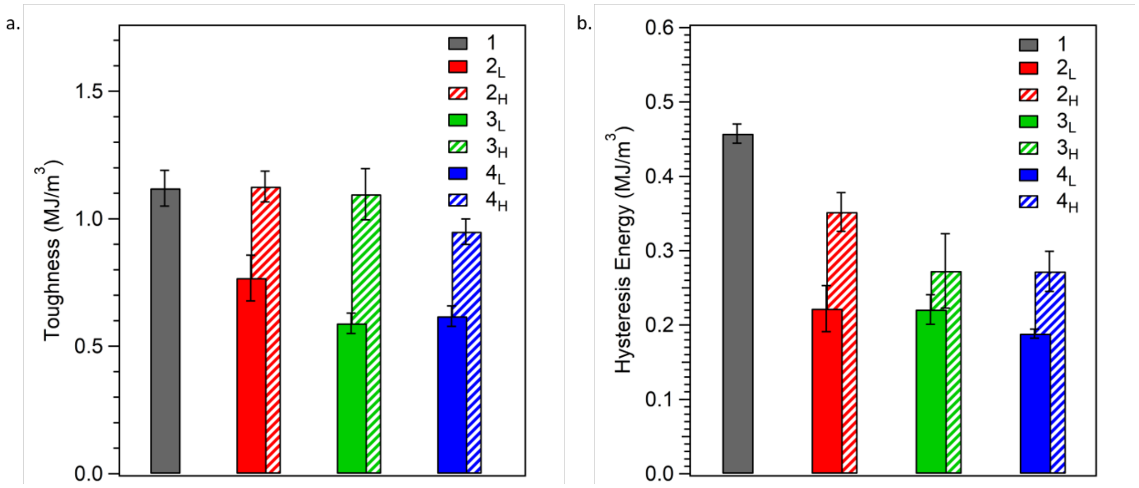

Fig. S7. Bar graphs showing (a) toughness or (b) hysteresis energy for the load/unload cycle for polymers stretched to 100% elongation. Values are an average of three measurements.

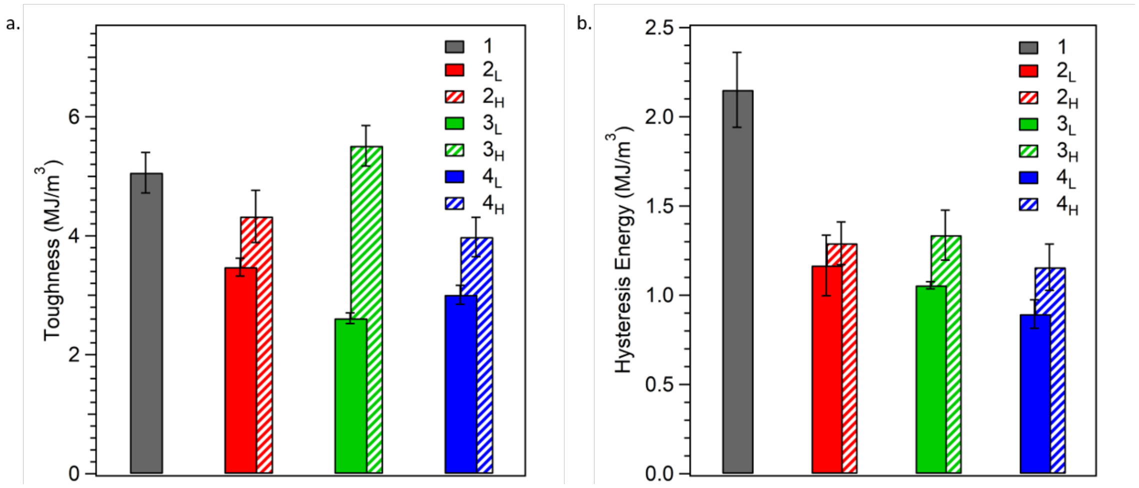

Fig. S8. Bar graphs showing (a) toughness or (b) hysteresis energy for the load/unload cycle for polymers stretched to 300% elongation**.** Values are an average of three measurements.

Table S5. Average hysteresis energy (W_H) and toughness (U_T) for loading/unloading cycle

Entry		$Đ$ PS Block 1	A_s PS Block 1	100% Strain W_H (MJ/m^3)	100% Strain U_T (MJ/m^3)	300% Strain W_H (MJ/m^3)	300% Strain U_T (MJ/m^3)	500% Strain W_H (MJ/m^3)	500% Strain U_T (MJ/m^3)
1	solid black line	1.06	1.62	0.5	1.1	2.2	5.1	5.5	11
2_L	solid red line	1.25	1.78	0.2	0.8	1.2	3.5	2.4	7.4
2_H	dotted red line	1.21	0.52	0.4	1.1	1.3	4.3	3.9	11
3_L	solid green line	1.43	2.41	0.2	0.6	1.1	2.6	2.9	6.3
3_H	dotted green line	1.54	0.35	0.3	1.1	1.3	5.5	3.5	12
4_L	solid blue line	1.63	3.37	0.2	0.6	0.9	3.0	2.4	6.9
4_H	dotted blue line	1.68	0.26	0.3	1.0	1.2	4.0	3.4	9.3

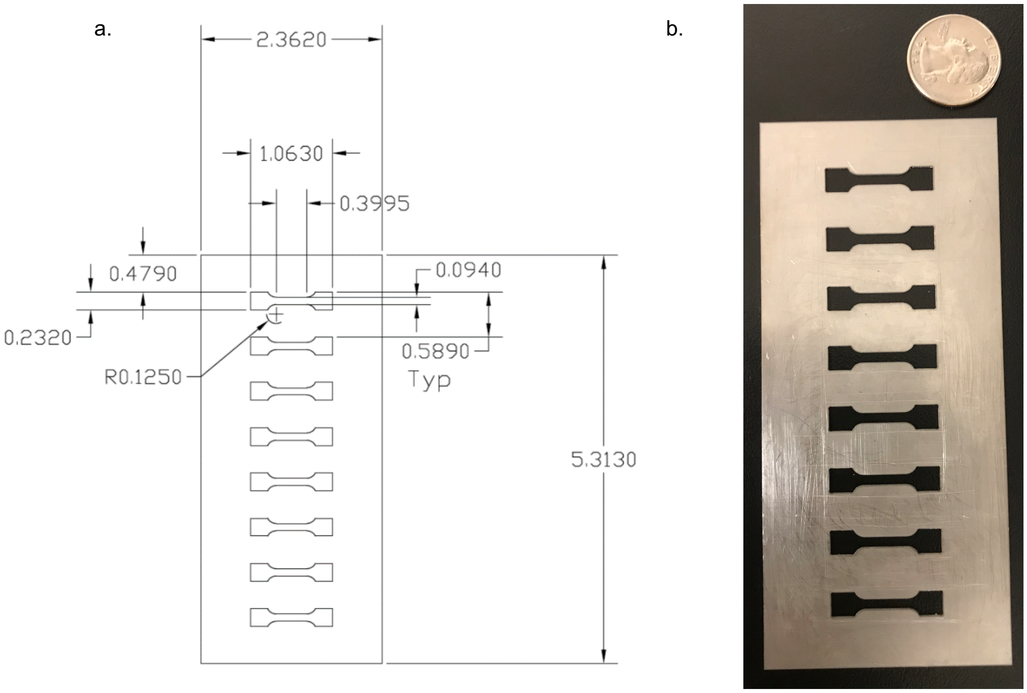

Fig. S9. (a) Specifications for stainless steel dog bone mould with plate thickness = 0.024”. Units are provided in inches. (b) Photograph of dog bone mould.

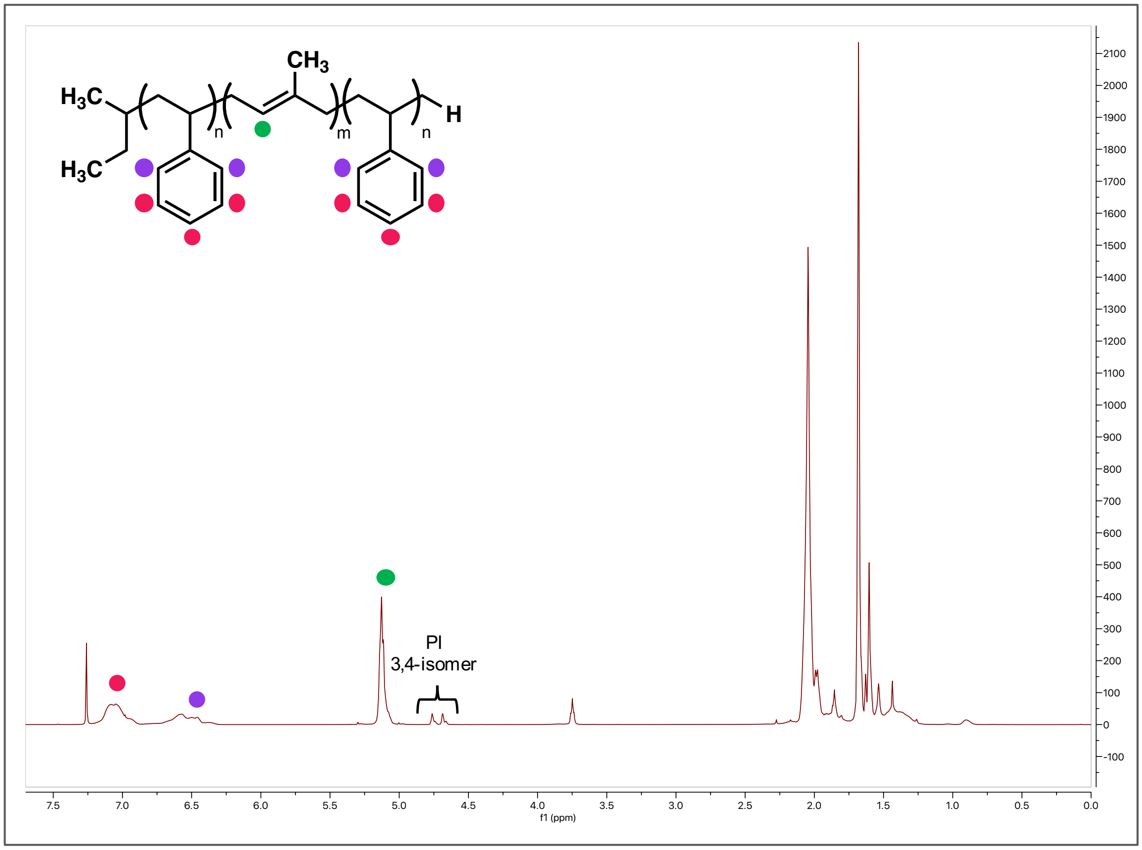

Fig. S10. Representative ^{1}H NMR of SIS triblock copolymer.

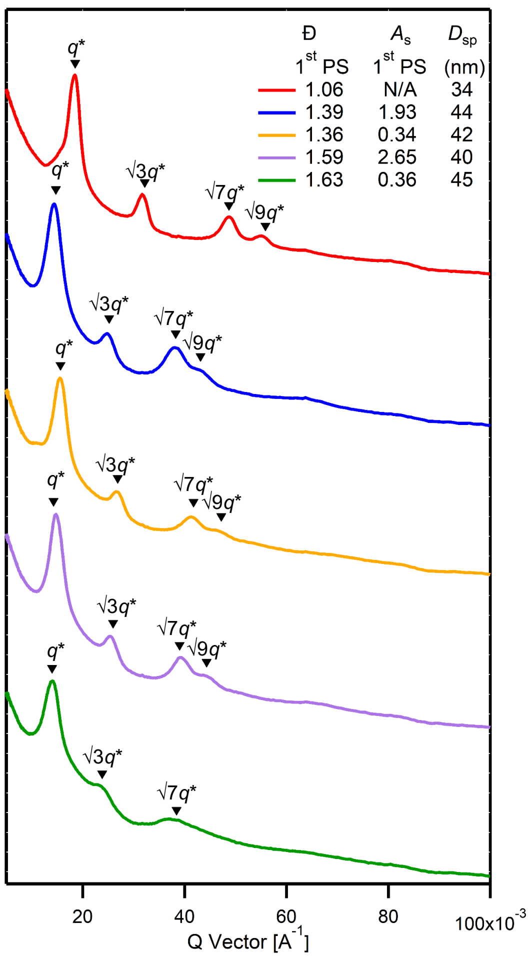

Fig. S11. Small-angle x-ray scattering (SAXS) traces of SIS samples with similar polymer composition and MWD features as those presented in the main text. Traces are offset for visual clarity. The volume fraction of PS is 0.22 in each sample, and the total molecular weights are as listed (top-to-bottom): 96, 101, 110, 109, and 109 kg/mol. Domain spacing was determined from the position of the principle wavevector (D_{sp} = $2\pi/q^*$). The indexed reflections indicate that all of the polymers share the same morphology of hexagonally packed cylinders.

Table S6. Stress Relaxation and Strain Recovery from Dynamic Mechanical Analysis

Entry		$Đ$ PS Block 1	A_s PS Block 1	Total Stress Relaxation (%)	Total Strain Recovery (%)
1	black solid line	1.06	1.62	55	53
2_L	red solid line	1.25	1.78	36	77
2_H	red dotted line	1.21	0.52	27	84
3_L	green solid line	1.43	2.41	46	65
3_H	green dotted line	1.54	0.35	21	86
4_L	blue solid line	1.63	3.37	38	85
4_H	blue dotted line	1.68	0.26	33	81

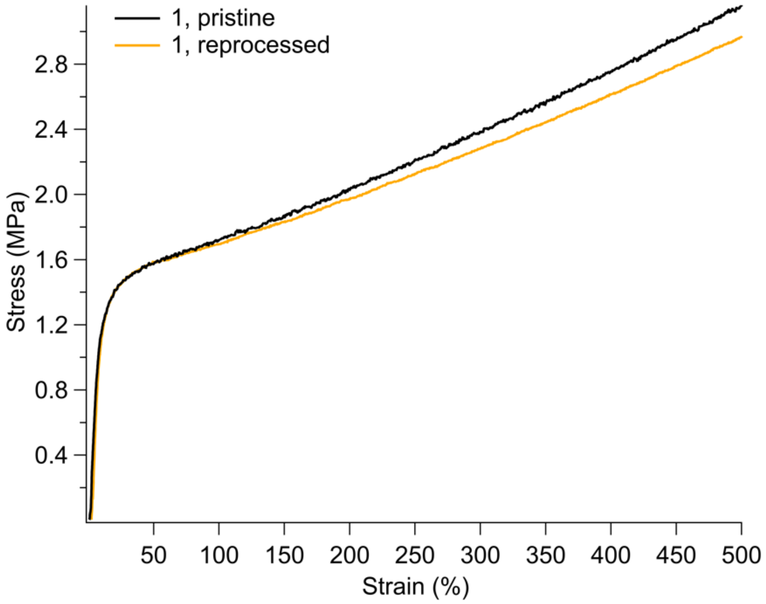

Fig. S12. Stress/strain curves of reference polymer **1** in pristine condition (black curve) and the same polymer after being reprocessed several times (orange curve).

References

1. Ilavsky, J. Nika: Softward for Two-Dimensional Data Reduction. *J. Appl. Cryst.* 2012, **45**, 324.
2. J. J Kirkland, W. W. Yau, H. J. Stoklosa, and C. H. J. Dilks, *J. Chromatogr. Sci.* 1977, **15**, 303-316.
